# Crotoxin Upregulating NLRP-3 Inflammasome and IL-18 and Activating CD4^+^ and CD8^+^ Lymphocytes in Experimental *Encephalitozoon cuniculi* Infection

**DOI:** 10.3390/ani16060955

**Published:** 2026-03-18

**Authors:** João Lourival de Souza, Eluane de Luca da Silva Martins, Anuska Marcelino Alvares Saraiva, Elizabeth Christina Perez, Ronalda Silva de Araújo, Sandra Coccuzzo Sampaio, Rodrigo Augusto Faganholi da Silva, Maria Anete Lallo

**Affiliations:** 1 Programa de Patologia Ambiental e Experimental, Universidade Paulista (UNIP), Rua Dr Bacelar 902, São Paulo 04026002, Brazil; joaolourivalutip@gmail.com (J.L.d.S.J.); eluanedelucas@gmail.com (E.d.L.d.S.M.); anuska.saraiva@docente.unip.br (A.M.A.S.); elizabeth.hurtado@docente.unip.br (E.C.P.); rodrigo.silva3@docente.unip.br (R.A.F.d.S.); 2 Departamento de Análises Ambientais, Divisão de Microbiologia e Parasitologia, Companhia Ambiental do estado de São Paulo (CETESB), Av. Prof. Frederico Hermann Júnior 345, São Paulo 05459900, Brazil; ronaldas.araujo@gmail.com; 3 Departmento de Fisiopatologia, Instituto Butantan, Av. Vital Brasil, 1500, Paulo 05503900, Brazil; sandra.coccuzzo@butantan.gov.br

**Keywords:** cytokines, crotoxin, inflammation, immunomodulation, microsporidia, zoonosis

## Abstract

*Encephalitozoon cuniculi* is an atypical, opportunistic, obligate intracellular fungal pathogen that infects vertebrates. It survives within the host by modulating the host immune response. Crotoxin (CTX), a bioactive compound isolated from the venom of *Crotalus durissus terrificus*, has been reported to modulate immune responses. This study evaluated the effects of CTX on the immune response of mice infected with *E. cuniculi*. Mice were immunosuppressed with cyclophosphamide (Cy), infected with *E. cuniculi* spores, and treated with a single dose of CTX on the day of experimental. The animals were euthanized on day 14 post-infection. Levels of T helper (Th1, Th2, and Th17) cytokines were measured in plasma, and macrophage and lymphocyte populations were analyzed in peritoneal lavage fluid and spleen. In addition, histopathological alterations, hepatic fungal burden, and mRNA expression levels of NLRP3 inflammasome–related genes were assessed. CTX upregulated NLRP3 inflammasome expression and increased IL-18 production, while reducing fungal burden in *E. cuniculi*-infected mice. Moreover, CTX increased the proportions of macrophages and B cells and enhanced IFN-γ expression in CD4^+^ and CD8^+^ T lymphocytes. Collectively, these findings indicate that CTX reduces fungal load in Cy-immunosuppressed mice infected with *E. cuniculi* by priming the NLRP3 inflammasome complex and upregulating IL-18 production.

## Simple Summary

*Encephalitozoon cuniculi* is an opportunistic intracellular fungal pathogen capable of causing severe infections in immunosuppressed hosts by evading immune defenses. Crotoxin, a bioactive molecule isolated from rattlesnake venom, has been reported to modulate immune responses. In the present study, we investigated whether crotoxin could enhance the immune response against *E. cuniculi* infection in immunosuppressed mice. Our results demonstrated that crotoxin treatment reduced the fungal burden and enhanced immune activation by stimulating the NLRP3 inflammasome and increasing IL-18 production. Additionally, crotoxin promoted the activation of macrophages, B cells, and both CD4^+^ and CD8^+^ T lymphocytes. Overall, these findings suggest that crotoxin may strengthen host immune defenses against opportunistic fungal infections.

## 1. Introduction

Inflammasomes are cytosolic multiprotein complexes that mediate the autocatalytic activation of inflammatory caspases, such as caspase-1 (CASP1), thereby regulating host defense through the induction of pyroptosis and the release of proinflammatory cytokines [1]. Among them, the NLRP3 inflammasome is the most broadly responsive to both microbial and sterile stimuli, acting as a key sensor of pathogen-associated molecular patterns (PAMPs), danger-associated molecular patterns (DAMPs), and environmental stress signals [2].

NLRP3 activation occurs through a two-step process: (i) a priming signal, typically initiated by NF-κB activation downstream of pattern recognition receptors (PRRs), which induces the transcription of *Nlrp3* and *pro–IL-1β*; and (ii) an activation signal triggered by infection or cellular stress, which promotes assembly of the inflammasome complex, autocatalytic cleavage of caspase-1, and the maturation of IL-1β and IL-18 [1,2,3]. Canonical activation requires the adaptor ASC, whereas noncanonical activation involves caspase-11 in mice (caspase-4 and -5 in humans), which sense cytosolic lipopolysaccharide and trigger gasdermin-mediated pore formation [4]. Both pathways ultimately culminate in pyroptosis, an inflammatory form of programmed cell death that eliminates intracellular niches and facilitates pathogen clearance [1,4]. Functionally, IL-1β promotes neutrophil recruitment and early pathogen control, whereas IL-18 enhances IFN-γ production and supports Th1-mediated antifungal immunity [1,4].

*Encephalitozoon cuniculi* is a unicellular, obligate intracellular microsporidian fungus that causes encephalitozoonosis—an emerging zoonotic and opportunistic infection of increasing medical and veterinary concern [5,6]. Microsporidia are ubiquitous in the environment and infect a broad range of hosts, including humans, livestock, and companion animals [7]. Their evolutionary success as intracellular pathogens is associated with their ability to manipulate host cell metabolism and evade immune recognition [8,9].

Experimental models employing pharmacological immunosuppression, such as cyclophosphamide (Cy) or dexamethasone, are widely used to reproduce the immunodeficient states observed in clinical microsporidiosis [10,11]. In Cy-treated, *E. cuniculi*-infected mice, susceptibility is characterized by reduced macrophage and lymphocyte counts, dysregulated cytokine profiles, and increased fungal burdens [12,13]. In vitro, *E. cuniculi* promotes macrophage polarization from a proinflammatory M1 phenotype toward an anti-inflammatory M2 phenotype, thereby generating a permissive environment for parasite replication [14].

The venom of *Crotalus durissus terrificus* contains several bioactive molecules with immunomodulatory properties. Among these, crotoxin (CTX)—a heterodimeric phospholipase A_2_ complex—has attracted considerable attention due to its regulatory effects on inflammation and immunity [15]. CTX inhibits lymphocyte proliferation and cytokine secretion, modulates macrophage migration and phagocytosis, and downregulates adhesion molecules and proinflammatory mediators [16,17,18,19]. Notably, CTX repolarizes *Leishmania amazonensis*-infected macrophages from an M2 phenotype toward a microbicidal M1 phenotype, enhancing nitric oxide and cytokine (IL-6, TNF-α) production and improving parasite clearance [20].

Recent evidence has shown that CTX lacks direct fungicidal activity against *E. cuniculi* spores but reprograms infected macrophages toward a proinflammatory M1 phenotype, increasing TNF-α and IL-6 secretion while reducing spore viability [21]. However, the molecular mechanisms underlying this immunomodulatory effect remain poorly understood. Given the pivotal role of the NLRP3 inflammasome in antifungal immunity, we hypothesized that CTX modulates inflammasome activation and cytokine maturation during *E. cuniculi* infection. Therefore, this study aimed to investigate the effects of CTX on NLRP3 inflammasome activation and IL-18 production in *E. cuniculi*-infected, immunosuppressed mice.

## 2. Materials and Methods

### 2.1. Ethics Agreement

All animal procedures were conducted in accordance with the guidelines established by the Brazilian National Council for the Control of Animal Experimentation (CONCEA) and approved by the Ethics Committee on Animal Use of Paulista University (protocol no. 9690131020).

### 2.2. Animals

Twenty-five (n = 25) specific pathogen-free (SPF) Balb/c mice (8–12 weeks old) were obtained from the Centro de Desenvolvimento de Modelos Experimentais (CEDEME, UNIFESP, São Paulo, Brazil). Animals were housed in microisolator cages at the Animal Experimentation Laboratory of Universidade Paulista under controlled conditions: a 12 h light/dark cycle, ambient temperature of 22–24 °C, relative humidity of 45–55%, with ad libitum access to irradiated chow and autoclaved water.

### 2.3. Encephalitozoon Cuniculi Spores

*E. cuniculi* spores (strain I; Waterborne Inc., New Orleans, LA, USA) were propagated in rabbit kidney-13 (RK-13; ATCC CCL-34) cells cultured in RPMI-1640 medium (Thermo Fisher Scientific, Waltham, MA, USA) supplemented with 10% fetal bovine serum (FBS; Gibco, Waltham, MA, USA) and 1% penicillin-streptomycin (Gibco). Cultures were maintained at 37 °C in a 5% CO_2_ atmosphere. Spores were harvested, washed three times with phosphate-buffered saline (PBS, pH 7.2), counted in a Neubauer chamber, and stored at 4 °C until use.

### 2.4. Cyclophosphamide-Induced Immunosuppression

Mice were intraperitoneally administered cyclophosphamide (Cy; Genuxal^®^, Asta Médica Oncologia, São Paulo, Brazil) at a dose of 200 mg/kg body weight, as previously described [10]. Two doses were administered before pathogen inoculation and two doses after infection to establish a pharmacological immunosuppression model consistent with microsporidiosis studies [10,12,13].

### 2.5. Crotoxin (CTX) Preparation and Treatment

#### 2.5.1. Purification of CTX

CTX was purified from the crude venom of *Crotalus durissus terrificus* following the procedure described by Rangel et al. [17], with minor modifications. Briefly, 10 mg of crude venom were dissolved in 2 mL of Tris-HCl buffer (50 mM, pH 7.3) and centrifuged at 10,000× *g* for 10 min (Eppendorf ultracentrifuge) to remove insoluble material. The supernatant was subjected to anion-exchange chromatography on a MONO-Q HR 5/5 column (5 mL) using an FPLC system (Fast Protein Liquid Chromatography, Pharmacia) equilibrated with Tris-HCl buffer (50 mM, pH 7.3). Bound proteins were eluted with a linear gradient of 0–1 M NaCl in the same buffer.

Three major protein peaks (I, II, and III) were detected, with Peak II corresponding to CTX. One-milliliter fractions were collected, and protein elution was monitored by absorbance at 280 nm. Fractions containing CTX were analyzed for homogeneity by SDS–polyacrylamide gel electrophoresis (SDS-PAGE) and tested for phospholipase A_2_ activity using a chromogenic substrate. The CTX-containing fractions were pooled, concentrated using a Vivaspin centrifugal (Vivaproducts, Great Road Littleton, MA, USA) concentrator, and quantified by the Bradford method.

#### 2.5.2. SDS-PAGE Electrophoretic Analysis for CTX Purity Assessment

The purity of the CTX fraction was evaluated by sodium dodecyl sulfate–polyacrylamide gel electrophoresis (SDS–PAGE). A 12.5% resolving gel was prepared and assembled between 8 × 5 cm glass plates with 1.0 mm spacers using the Mini-PROTEAN II System (Bio-Rad, Hercules, CA, USA). Venom and CTX samples were diluted in sample buffer containing 125 mM Tris-HCl (pH 6.9), 2.5% (*w*/*v*) SDS, 20% glycerol, 1 mM PMSF, 4 mM EDTA, and 0.05% bromophenol blue, and then denatured by heating at 100 °C for 5 min.

Samples were loaded onto a 4% stacking gel and electrophoresed at a constant current of 20 mA for approximately 2 h. After electrophoresis, protein bands were visualized by Coomassie Brilliant Blue staining. To ensure sample safety for subsequent in vivo use, CTX preparations were tested for endotoxin (LPS) contamination using a Limulus Amebocyte Lysate (LAL) chromogenic assay (Cambrex/Lonza, East Rutherford, NJ, USA), performed at the Quality Control Microbiology Laboratory of the Bioindustrial Center, Butantan Institute (São Paulo, Brazil). CTX aliquots confirmed to be endotoxin-free were stored at −20 °C until use.

#### 2.5.3. Administration Protocol

A single subcutaneous dose of CTX (44 μg/kg body weight) was administered in the dorsal region simultaneously with *E. cuniculi* inoculation on day 0. This dosage was selected based on previous studies showing immunomodulatory effects without inducing clinical signs of envenomation [19,20]. 

### 2.6. Experimental Design

**Figure 1:**
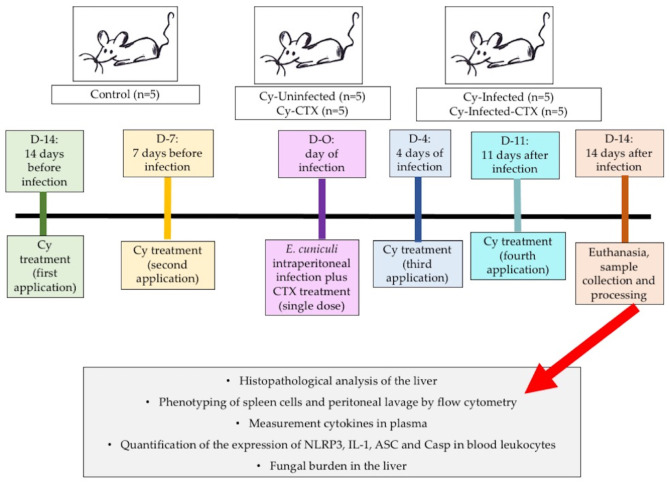
Experimental design. BALB/c mice were randomly assigned to five groups (n = 5 per group): Control—untreated and uninfected; Cy—received cyclophosphamide only; Cy + CTX—received cyclophosphamide and crotoxin; Cy + Infection—received cyclophosphamide and were infected with *Encephalitozoon cuniculi*; and Cy + Infection + CTX—received cyclophosphamide, were infected with *E. cuniculi*, and treated with crotoxin. Cyclophosphamide was administered in four doses (two before and two after infection). Crotoxin (44 μg/kg body weight) was given as a single dose on the day of experimental infection. Fourteen days post-infection, animals were weighed and euthanized. Whole blood was collected via cardiac puncture to obtain plasma for cytokine quantification and *Nlrp3* inflammasome gene expression analysis in circulating leukocytes. Peritoneal lavage fluid was used for macrophage and lymphocyte phenotyping, the spleen was mechanically dissociated for immune cell analysis, and liver samples were collected for histopathological evaluation and fungal burden quantification.

Mice were randomly assigned to five groups (n = 5 per group): Control (untreated and uninfected); Cy (received cyclophosphamide only); Cy + CTX (received cyclophosphamide and crotoxin); Cy + Infection (received cyclophosphamide and were infected with *E. cuniculi*); and Cy + Infection + CTX (received cyclophosphamide, were infected with *E. cuniculi*, and were treated with CTX) (Figure 1).

Immunosuppression was maintained by administering cyclophosphamide once weekly for four weeks (two doses prior to infection and two doses post-infection). Mice were infected intraperitoneally with 1 × 10^7^ *E. cuniculi* spores (in 0.2 mL volume) and treated with CTX as described above. On day 14 post-infection, animals were weighed and euthanized under deep anesthesia induced with ketamine, xylazine, and acepromazine. Blood, peritoneal lavage fluid, spleen, and liver samples were collected for subsequent analyses, as described below.

### 2.7. Necropsy, Sample Collection and Histopathological Analysis

After euthanasia, blood was collected via cardiac puncture into EDTA-coated tubes, centrifuged (200 g, 10 min), and plasma stored at −80 °C for cytokine analysis. Leukocytes were isolated and preserved at −80 °C for RNA extraction. Peritoneal lavage was performed using 10 mL of PBS containing 1% BSA and 0.75% EDTA. Spleens were mechanically dissociated through 70 µm strainers and erythrocytes lysed with hemolytic buffer. Liver lobes were divided for fungal load quantification (−80 °C storage) and histopathological processing (10% buffered formalin, 72 h fixation). Formalin-fixed, paraffin-embedded liver sections (4 µm) were stained with hematoxylin and eosin (H&E) and examined under a Leica DMLD light microscope (Leica Microsystems, Bufallo Grove, IL, USA). Inflammatory infiltrates, necrosis, and granulomatous lesions were semi-quantitatively scored by blinded observers using standardized criteria.

### 2.8. Flow Cytometry

Cells were stained with fluorochrome-conjugated monoclonal antibodies (mAbs) against specific surface markers (Table 1). For intracellular staining, cells were fixed and permeabilized using the BD Cytofix/Cytoperm kit (BD Biosciences, Milpitas, CA, USA) according to the manufacturer’s protocol. Data were acquired on a BD Accuri™ C6 cytometer and analyzed using FlowJo v10.0 (BD Biosciences, USA). Gating strategies were validated by fluorescence-minus-one (FMO) controls (Supplementary Figure S1). Flow cytometry data were acquired using identical instrument settings, and mean fluorescence intensity (MFI) values were calculated using the same gating strategy across all samples.

### 2.9. Cytokine Quantification

Plasma cytokines were quantified using the BD CBA Mouse Th1/Th2/Th17 Cytokine Kit (BD Biosciences, USA) for IL-2, IL-4, IL-6, IL-10, IL-17A, TNF, and IFN-γ, following manufacturer’s guidelines. Samples were acquired on a BD Accuri™ C6 and analyzed using FCAP Array software v3.0 (BD Biosciences, USA).

### 2.10. NLRP3 Inflammasome Gene Expression

#### 2.10.1. RNA Extraction and Quality Assessment

Total RNA was extracted from leukocytes using TRIzol^®^ reagent (Invitrogen, Carlsbad, CA, USA), followed by chloroform/isopropanol precipitation. RNA purity and concentration were assessed by spectrophotometry (NanoDrop 2000, Thermo Fisher Scientific, USA) based on OD260/280 (≥1.8) and OD260/230 (≥1.0) ratios. Samples were stored at −80 °C.

#### 2.10.2. Complementary DNA (cDNA) Synthesis

Two micrograms of total RNA were reverse transcribed into cDNA using the SuperScript II Reverse Transcriptase Kit (Invitrogen, USA) in a 20 μL reaction containing dNTPs, Oligo(dT) primers, and RNase inhibitor. The reaction was incubated at 50 °C for 50 min and terminated at 85 °C for 5 min. The resulting cDNA was diluted to 100 ng/μL and stored at −20 °C.

#### 2.10.3. Quantitative Real-Time PCR (qRT-PCR)

Gene expression was quantified by qRT-PCR using SYBR Green Master Mix (Roche, Indianapolis, IN, USA) on a QuantStudio™ 5 Real-Time PCR System (Applied Biosystems, Carlsbad, CA, USA). Primers used are listed in Table 2. Expression levels were normalized to *Rnu6* as the reference gene, and relative expression was calculated using the 2^−ΔCt^ method. All reactions were performed in duplicate, and results represent means ± SD from five biological replicates.

### 2.11. Quantification of Fungal Burden

Fungal load in liver tissue was determined by qRT-PCR targeting the *E. cuniculi* 18S rRNA gene (GenBank: X98470.1), as described previously [12]. DNA was extracted using the DNeasy Blood and Tissue Kit (Qiagen, Hilden, Germany). Standard curves were constructed using serial dilutions of synthetic gBlock^®^ gene fragments (IDT, San Diego, CA, USA) ranging from 2.8 × 10^8^ to 2.8 × 10^2^ gene copies/μL. Copy numbers were calculated from the standard curve and expressed as spores per mg of liver tissue, assuming 22 copies of 18S rRNA per genome.

### 2.12. Statistical Analysis

Data are presented as mean ± SEM. Statistical analyses were performed using one-way ANOVA followed by Tukey’s post hoc test or Student’s *t*-test when appropriate, using GraphPad Prism v5.0 (GraphPad Software, Boston, MA, USA). Statistical significance was defined *p* < 0.05 *, *p* < 0.01 **, *p* < 0.001 ***, *p* < 0.0001 ****.

## 3. Results

### 3.1. CTX Promotes Pathogen Control and Mitigates Clinical Signs

**Figure 2:**
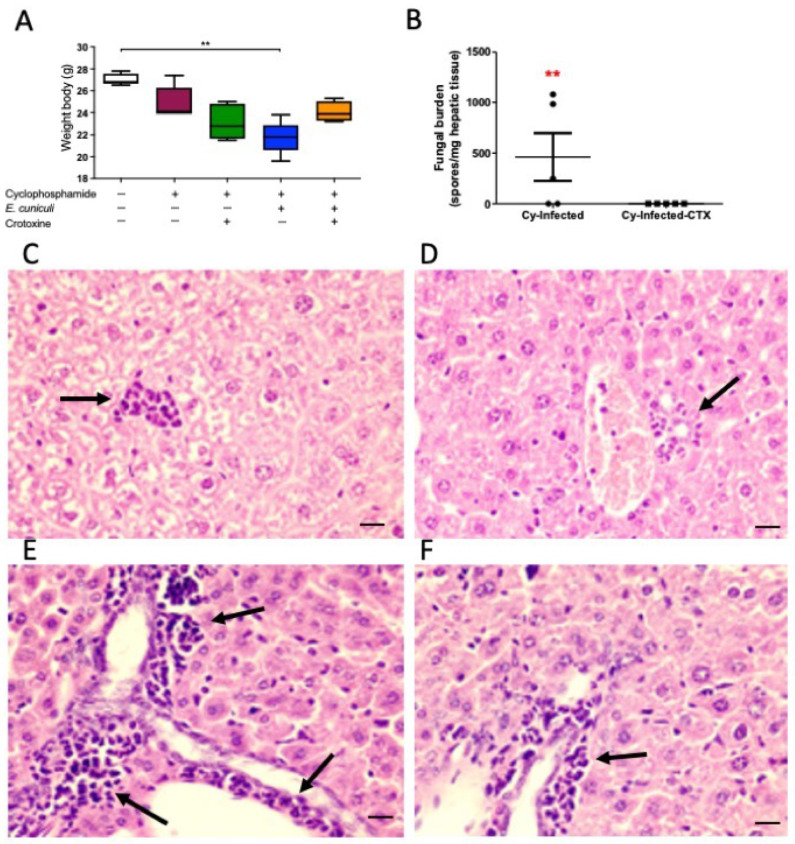
Changes in clinical, fungal burden, and histopathological parameters in experimental encephalitozoonosis. (**A**) Mean body weight (g) of animals from different groups treated (+) or not (−) with cyclophosphamide (Cy) and/or crotoxin (CTX) and infected (+) or not (−) with *Encephalitozoon cuniculi*. One-way ANOVA followed by Tukey’s post hoc test; *p* < 0.01 (**). (**B**) Comparison of fungal burden determined by quantitative PCR in the Cy + Infection and Cy + Infection + CTX groups (*t*-test). (**C**) Liver parenchyma of Cy-treated, uninfected mice showing discrete leukocytic infiltrate (arrow). (**D**) Liver parenchyma of Cy + CTX + uninfected mice showing mild periportal neutrophilic infiltrate (arrow). (**E**) Liver parenchyma of Cy + Infection mice showing marked portal leukocytic infiltration (arrow). (**F**) Liver parenchyma of Cy + Infection + CTX mice showing portal leukocytic infiltration (arrow). Hematoxylin and eosin (H&E) staining, Barr = 10 μm.

Cyclophosphamide (Cy)–treated mice exhibited approximately 10% body weight loss relative to controls, consistent with systemic immunosuppression (Figure 2A). Mice receiving CTX alone showed a 14% reduction in body weight, indicating a physiological response to CTX administration. The Cy + Infection group experienced the greatest weight loss (~21%) and presented with ruffled fur and reduced activity, reflecting heightened infection susceptibility under immunosuppression. No mortality occurred in any experimental group throughout the observation period.

Quantitative real-time PCR targeting *E. cuniculi* genomic DNA in liver samples revealed undetectable fungal burden in the Cy + Infection + CTX group (Figure 2B), indicating that CTX treatment effectively eliminated the pathogen. Histopathological evaluation of liver tissue—an early target organ following intraperitoneal inoculation—showed mild neutrophilic infiltration in animals treated with Cy and/or CTX (Figure 2C,D). In contrast, infected groups displayed mononuclear cell infiltrates within the hepatic parenchyma and periportal regions (Figure 2E,F). Although infection induced a distinct inflammatory profile, no significant histological differences were detected between infected mice treated with or without CTX.

### 3.2. CTX Enhances NLRP3 Inflammasome Priming and Increases Systemic IL-18

**Figure 3:**
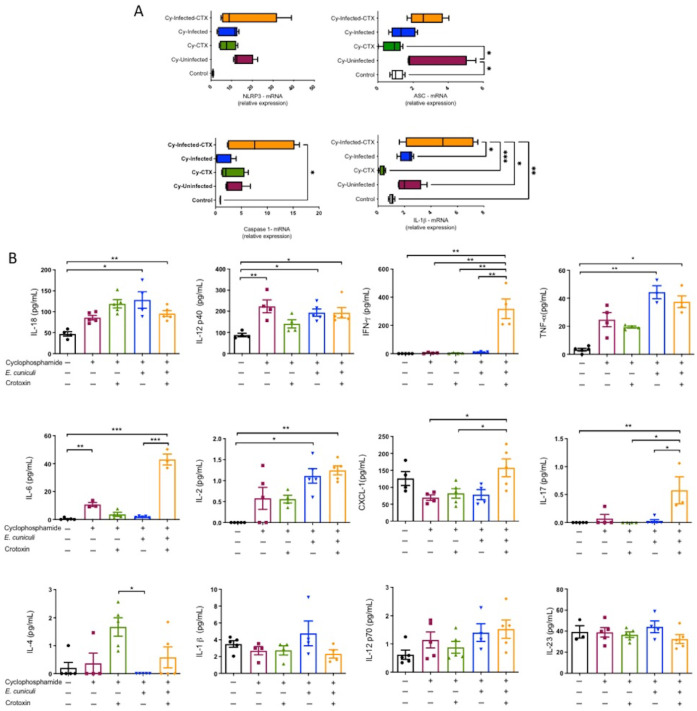
Cytokine inflammatory profile and activation of the NLRP3 inflammasome. (**A**) Relative mRNA expression of *Nlrp3*, *Asc*, *Caspase-1*, and *IL-1β* in circulating leukocytes from the following groups: Control—untreated and uninfected; Cy—treated with cyclophosphamide only; Cy + CTX—treated with cyclophosphamide and crotoxin; Cy + Infection—treated with cyclophosphamide and infected with *Encephalitozoon cuniculi*; and Cy + Infection + CTX—treated with cyclophosphamide, infected with *E. cuniculi*, and treated with crotoxin. (**B**) Plasma levels of cytokines IL-18, IL-12p40, IFN-γ, TNF-α, IL-6, IL-2, CXCL1, IL-17, IL-1β, IL-12p70, IL-23, and IL-4 in mice treated (+) or not (−) with cyclophosphamide and/or crotoxin and infected (+) or not (−) with *E. cuniculi*. One-way ANOVA followed by Tukey’s post hoc test, *p* < 0.05 *, *p* < 0.01 **, *p* < 0.001 ***.

To further explore molecular changes associated with pathogen control, expression levels of NLRP3 inflammasome–related genes were quantified in peripheral leukocytes. qRT-PCR revealed significant upregulation of Nlrp3, Asc, Casp1, and IL-1β transcripts in the Cy + Infection + CTX group compared with all other groups (Figure 3A), supporting enhanced transcriptional priming of the inflammasome pathway. Consistent with this, plasma IL-18 concentrations were markedly elevated in the Cy + Infection + CTX group, whereas IL-1β levels did not differ significantly among treatments (Figure 3B).

Comprehensive cytokine profiling demonstrated a pronounced Th1-skewed response: IFN-γ levels were approximately tenfold higher in Cy + Infection + CTX than in Cy + Infection (Figure 3B), which is compatible with IL-18–associated amplification of IFN-γ production. Elevated levels of IL-6, CXCL1, and IL-17 were also observed in both infected groups, while IL-4 increased selectively in Cy + Infection + CTX, suggesting concurrent pro- and anti-inflammatory modulation (Figure 3B).

### 3.3. CTX Potentiates T-Cell IFN-γ Responses Under Lymphodepletion

**Figure 4:**
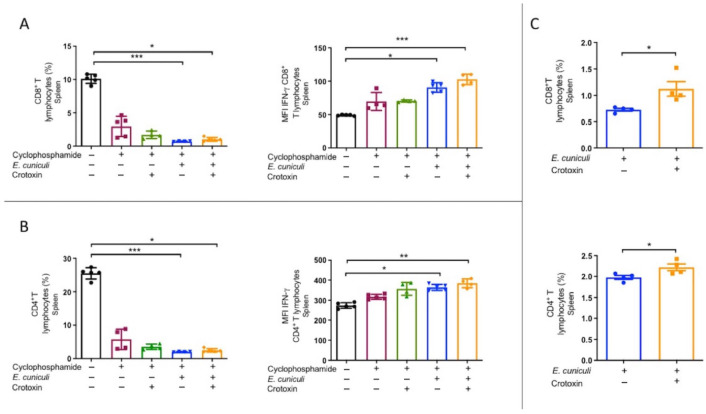
Reduction in T lymphocyte populations and increased activation. Evaluation of splenic T lymphocytes from mice treated (+) or not (−) with cyclophosphamide and/or crotoxin and inoculated (+) or not (−) with *Encephalitozoon cuniculi*. (**A**) Frequency of CD8^+^ T lymphocytes and median fluorescence intensity (MFI) of IFN-γ within CD8^+^ T cells. (**B**) Frequency of CD4^+^ T lymphocytes and MFI of IFN-γ within CD4^+^ T cells. (**C**) Comparison of CD8^+^ and CD4^+^ T-cell percentages between *E. cuniculi*-infected animals treated or not with crotoxin. One-way ANOVA followed by Tukey’s post hoc test (**A**,**B**) or *t*-test (**C**); *p* < 0.05 *, *p* < 0.01 **, *p* < 0.001 ***.

Flow cytometric analysis of splenic lymphocytes revealed that both CD4^+^ and CD8^+^ T-cell frequencies were reduced across all Cy-treated groups compared with controls (Figure 4). Despite this lymphodepletion, the median fluorescence intensity (MFI) of intracellular IFN-γ was significantly elevated in both Cy + Infection and Cy + Infection + CTX groups (Figure 4A–C). The increase was most pronounced in Cy + Infection + CTX, indicating that CTX enhances T-cell effector function even under immunosuppressive conditions.

### 3.4. Infection and Cy/CTX Treatment Favor an M2-like Peritoneal Macrophage Phenotype

**Figure 5:**
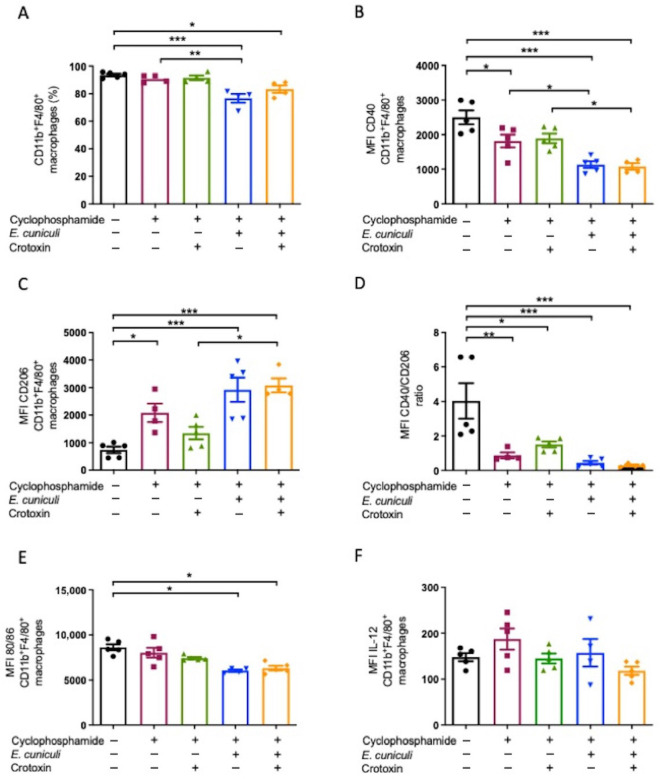
Increase in the M2 macrophage profile following treatment with Cy, CTX, or infection with *Encephalitozoon cuniculi*. (**A**) Frequency (%) of total CD11b^+^F4/80^+^ macrophages in mice treated (+) or not (−) with cyclophosphamide (Cy) and/or crotoxin and inoculated (+) or not (−) with *E. cuniculi*. (**B**) Median fluorescence intensity (MFI) of CD40 in CD11b^+^F4/80^+^ macrophages. (**C**) MFI of CD206 in CD11b^+^F4/80^+^ macrophages. (**D**) Ratio of CD40/CD206 MFI in CD11b^+^F4/80^+^ macrophages. (**E**) MFI of co-stimulatory molecules CD80 and CD86 in CD11b^+^F4/80^+^ macrophages. (**F**) Percentage (%) of CD11b^+^F4/80^+^ macrophages positive for IL-12A. One-way ANOVA followed by Tukey’s post hoc test, *p* < 0.05 *, *p* < 0.01 **, *p* < 0.001 ***.

Flow cytometry of peritoneal exudate cells demonstrated a reduction in CD11b^+^F4/80^+^ macrophages in Cy + Infection and Cy + Infection + CTX compared with controls (Figure 5A). The M1-associated marker CD40^+high^ was downregulated, while the M2-associated marker CD206^+high^ was upregulated during infection, irrespective of CTX treatment (Figure 5B,C). In both Cy and Cy + CTX groups, CD40 MFI remained lower than CD206 MFI (Figure 5D).

Additionally, macrophages from Cy and Cy + Infection + CTX groups exhibited reduced expression of co-stimulatory molecules CD80 and CD86 (Figure 5E), while IL-12 production remained unchanged across groups (Figure 5F). These results indicate a predominant M2-like immunosuppressive phenotype in the peritoneal compartment under Cy and CTX influence. No significant changes in splenic macrophage subsets were detected (Supplementary Figure S2).

### 3.5. Cy Reduces B-Cell Subsets, While B-1b Cells Predominate During Infection

**Figure 6:**
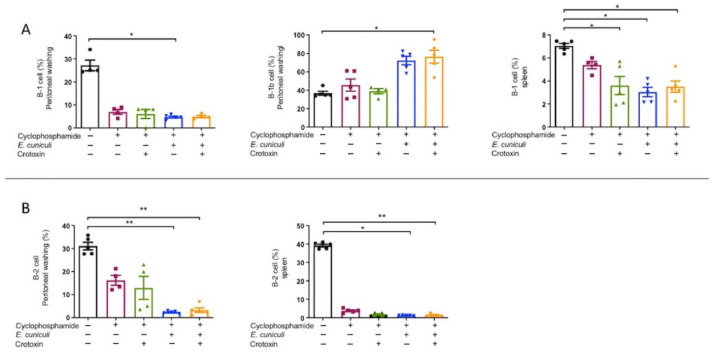
Evaluation of B-cell populations in peritoneal lavage and spleen. Analysis of B cells from mice treated (+) or not (−) with cyclophosphamide (Cy) and/or crotoxin and inoculated (+) or not (−) with *Encephalitozoon cuniculi*. (**A**) Frequency (%) of B-1 (CD19^+^CD23^−^) lymphocytes in peritoneal lavage and spleen, and of B-1b (CD19^+^CD23^−^CD5^−^) lymphocytes in peritoneal lavage. (**B**) Frequency (%) of B-2 (CD19^+^CD23^+^) lymphocytes in peritoneal lavage and spleen. One-way ANOVA followed by Tukey’s post hoc test, *p* < 0.05 *, *p* < 0.01 **.

Cyclophosphamide markedly decreased both B-1 and B-2 cell populations in the peritoneum and spleen (Figure 6A,B). In infected animals (Cy + Infection and Cy + Infection + CTX), the B-1b subset represented approximately 60–95% of peritoneal B cells (Figure 6A), suggesting a compensatory role for B-1b cells in antigen presentation in response to macrophage depletion.

## 4. Discussion

This study demonstrates that CTX markedly reduces fungal burden in *Encephalitozoon cuniculi*–infected mice, indicating a potent anti-microsporidial effect mediated through immunomodulation. The mechanistic basis of this effect appears to involve the upregulation of genes associated with the NLRP3 inflammasome complex and increased systemic IL-18 levels, supporting a model in which CTX enhances innate immune activation and thereby promotes pathogen clearance.

Inflammasomes are multiprotein complexes activated by microbial infections or cellular stress that mediate caspase-1–dependent maturation of IL-1β and IL-18. Among them, the NLRP3 inflammasome—composed of NLRP3, ASC, and caspase-1—is widely expressed in myeloid cells such as neutrophils, monocytes, and dendritic cells and can be triggered by diverse microbial stimuli. Once activated, caspase-1 promotes the maturation of IL-1β and IL-18 and induces pyroptosis, an inflammatory form of cell death that contributes to pathogen elimination [22,23,24].

The present findings provide, for the first time, in vivo evidence that CTX transcriptionally primes the NLRP3 inflammasome pathway and elevates IL-18 levels in infected, immunosuppressed mice. These molecular events were associated with reduced fungal load and attenuated clinical signs, suggesting that CTX contributes to pathogen control by reprogramming the inflammatory milieu. Corroborating previous data from Nascimento de Oliveira (2025) [21], who reported that CTX induces M1 polarization and enhances microbicidal activity in *E. cuniculi*–infected macrophages, our results further demonstrate that CTX upregulates *Nlrp3*, *Asc*, *Casp1*, and IL-1β mRNA expression, leading to enhanced IL-18 secretion. Taken together, these findings indicate that CTX promotes a proinflammatory environment consistent with enhanced inflammasome priming, which may favor canonical NLRP3 signaling and fungal control.

At the cellular level, CTX induced distinct immunological adjustments. Despite a general decrease in macrophage, B-cell, and T-cell populations—likely attributable to cyclophosphamide-induced myelosuppression—CTX significantly increased IFN-γ expression in CD4^+^ and CD8^+^ T cells. This suggests that CTX compensates for quantitative immune suppression by enhancing qualitative effector activation. Notably, IL-18 is a potent co-stimulatory cytokine for IFN-γ production, acting synergistically with IL-12 or IL-15 to enhance IL-18 receptor (IL-18Rα) expression. Elevated IL-18 may therefore underlie the observed increase in IFN-γ–producing T cells, contributing to pathogen control via enhanced Th1 immunity. In addition, IL-18 amplifies the cytotoxic potential of NK and CD8^+^ T cells, promoting target-cell lysis through perforin or Fas–FasL–mediated apoptosis [24,25].

Consistent with this model, plasma cytokine profiling revealed a coordinated upregulation of IL-18, TNF-α, IFN-γ, IL-2, IL-12p40, and IL-6 in Cy + Infection and Cy + Infection + CTX groups. Cyclophosphamide itself is known to modulate redox metabolism, generating reactive oxygen species (ROS) that activate transcription factors such as NF-κB, thereby stimulating the expression of proinflammatory mediators including IL-6, TNF-α, and COX-2 [26,27]. Previous studies have reported similar elevations of IFN-γ, TNF, and IL-6 in cyclophosphamide-immunosuppressed mice infected with *E. cuniculi* [12,13], aligning with our data. Moreover, Moschella et al. (2011) [28] demonstrated that cyclophosphamide upregulates genes belonging to the IL-1 family, including *IL-1b*, *IL-18*, and IFN-γ *-*. In the current study, the concomitant increase in IL-18 and inflammasome-related gene expression in CTX-treated infected mice reinforces the view that IL-18 synergizes with IL-12 to activate NK and T cells, thereby sustaining IFN-γ production [29]. The observed coordination between inflammasome activation, IL-18 secretion, and Th1 cytokine upregulation likely represents a central mechanism of resistance to *E. cuniculi* infection.

It is important to note that cyclophosphamide itself is known to modulate host immune responses and inflammatory pathways, including alterations in immune cell populations and cytokine production. Previous studies using cyclophosphamide-immunosuppressed mice infected with *E. cuniculi* have reported increased fungal burden and enhanced susceptibility to infection, reflecting impaired cellular immunity under immunosuppressive conditions. In this context, the reduction in fungal burden observed in CTX-treated mice suggests that CTX exerts additional immunomodulatory effects that may enhance antimicrobial defense mechanisms despite cyclophosphamide-induced immune suppression.

Macrophage polarization analysis revealed that *E. cuniculi* infection drives a shift toward an M2-like phenotype under immunosuppressive conditions. The predominance of CD206^+^high and the reduction in CD40^+^high, CD80^+^, CD86^+^, and IL-12 expression in macrophages from infected or CTX-treated animals indicate an anti-inflammatory bias. This is consistent with prior observations in other parasitic models. For example, in *Leishmania amazonensis* infection, CTX enhanced macrophage phagocytic capacity and intracellular parasite killing through nitric oxide production and M1 polarization [20]. Conversely, bone marrow–derived macrophages from septic mice treated with CTX exhibited decreased microbicidal activity and an M2-like phenotype [30]. Importantly, macrophage polarization represents a functional continuum rather than a fixed binary state. The persistence of an M2-like surface marker profile does not necessarily preclude the activation of specific inflammatory pathways or coordinated immune responses. In the present study, despite the predominance of an M2-like macrophage phenotype, CTX-treated mice exhibited significantly reduced fungal burden. This observation suggests that pathogen control may not rely exclusively on macrophage repolarization but rather on integrated immune mechanisms.

Increased IL-18 levels and a pronounced Th1-skewed cytokine response, particularly elevated IFN-γ, indicate that CTX may potentiate antifungal immunity through crosstalk between innate and adaptive immune compartments. IL-18–dependent amplification of IFN-γ production by T cells and NK cells could compensate for macrophage anti-inflammatory polarization and enhance overall pathogen control. Thus, CTX-mediated protection likely reflects multicellular immune modulation rather than direct M2-to-M1 repolarization. Further studies are required to elucidate the precise cross-regulatory mechanisms involved.

Regarding lymphocyte dynamics, CTX is known to suppress splenic T-cell proliferation and IL-4 production in antigen-immunized mice [31]. Similarly, Zambelli et al. (2008) [19] reported that CTX decreases circulating lymphocytes while promoting T (CD3^+^) and B (CD45R^+^) cell accumulation in mesenteric lymph nodes. In the present study, cyclophosphamide decreased CD4^+^ and CD8^+^ T-cell frequencies; however, CTX treatment restored T-cell activation and elevated IFN-γ expression. These phenotypes suggest that CTX counteracts the immunosuppressive effects of cyclophosphamide by enhancing Th1 effector responses, thereby improving pathogen clearance.

Cyclophosphamide, a cytotoxic alkylating agent metabolized by cytochrome P450 enzymes, suppresses hematopoietic cell division and induces apoptosis, leading to lymphopenia and altered immune cell composition [27,28]. In agreement with this, our data show reduced B-1 and B-2 cell populations in the spleen and peritoneum of Cy-treated mice. B cells, particularly the B-1 subset, play crucial roles in early defense against *E. cuniculi* infection. Prior studies demonstrated that X-linked immunodeficient (XID) mice lacking B-1 and B-2 cells exhibit heightened susceptibility to *E. cuniculi* [32,33], resembling the dissemination pattern observed in SCID or nude mice. Consequently, the observed depletion of B cells in Cy-treated groups likely contributes to increased susceptibility and enhanced inflammatory infiltration.

Interestingly, although total B-1 cells were reduced, the B-1b subset predominated among residual lymphocytes in infected mice, representing 60–95% of peritoneal B cells. B-1b cells, unlike CD5^+^ B-1a cells, have enhanced antigen-presenting capabilities and serve as a bridge between innate and adaptive immunity [34]. The expansion of this subset may therefore represent a compensatory mechanism to sustain antigen presentation in the face of reduced macrophage numbers.

Collectively, these results establish that CTX exerts a multifaceted immunomodulatory effect during *E. cuniculi* infection, characterized by (i) transcriptional priming of the canonical NLRP3 inflammasome, (ii) IL-18–mediated amplification of IFN-γ–dependent Th1 immunity, and (iii) modulation of macrophage and B-cell responses under immunosuppressive conditions. The integration of inflammasome activation with adaptive immune reinforcement positions CTX as a promising immunotherapeutic candidate for opportunistic microsporidial infections, particularly in immunocompromised hosts. Future work should dissect the relative contributions of IL-18, NK-cell activity, and macrophage reprogramming to the overall antifungal efficacy of CTX.

## 5. Conclusions

In summary, CTX treatment markedly reduced fungal burden in *Encephalitozoon cuniculi*–infected, cyclophosphamide-immunosuppressed mice. The protective effect of CTX was associated with transcriptional upregulation of components of the canonical NLRP3 inflammasome pathway and increased systemic IL-18 levels, suggesting enhanced inflammasome priming rather than direct evidence of full inflammasome activation. Although *E. cuniculi* infection alone induced a modest Th1 cytokine profile, it did not significantly alter the expression of inflammasome-related genes. These findings indicate that CTX-mediated immune modulation—rather than direct antifungal activity—likely contributes to the improved pathogen control observed.

These findings demonstrate that strategic modulation of host immunity can counteract intracellular pathogens that evade classical microbicidal mechanisms. Collectively, this study identifies CTX as a potent immunomodulatory agent capable of restoring antifungal defenses under immunosuppressive conditions. Further investigation into inflammasome-targeted interventions may provide new therapeutic avenues for the management of opportunistic infections such as encephalitozoonosis.

## Figures and Tables

**Table 1:** Specifications of antibodies used in flow cytometry.

Panels	Antibody	Conjugate	Company
Panel 1 for the characterization of B lymphocyte subpopulations	CD23	FITC	BD Biosciences
	CD19	PerCP	BD Biosciences
	CD11b	APC	BD Biosciences
	CD5	PE	BD Biosciences
Panel 2 for the characterization of macrophages and M1 and M2 macrophages	CD11b	FITC	BD Biosciences
	F4/80	PE	eBioscience (San Diego, CA, USA)
	CD40	PE/Cy5	BD Biosciences
	CD206	AF 647	eBioscience
Panel 3 for the characterization of macrophage activation	CD11b	PerCP/Cy5	BD Biosciences
	F4/80	PE	eBioscience
	CD80	FITC	eBioscience
	CD86	FITC	eBioscience
	IL-12 (p40/p70)	APC	BD Biosciences
Panel 4 for the characterization of T lymphocytes	CD4	PerCP-Cy5	BD Biosciences
	CD8	FITC	BD Biosciences
	IFNγ	APC-	eBioscience

**Table 2:** Cycling conditions and sequence of primers used to study gene expression.

Gene (ID)	Primers	5′-3′ Sequence	Reaction Conditions	Product Size (bp)
*Nrlp3* (11461)	Forward	ATT ACC CGC CCG AGA AAG G	95 °C—15 s; 58 °C—30 s, 72 °C—30 s	141
	Reverse	TCG CAG CAA AGA TCC ACA CAG		
*Asc1* (14433)	Forward	AGA CAT GGG CTT ACA GGA	95 °C—15 s; 60 °C—30 s, 72 °C—30 s	256
	Reverse	CTC CCT CAT CTT GTC TTG G		
*Caspase 1* (14433)	Forward	TGA AAG AGG TGA AAG AAT T	95 °C—15 s; 59 °C—30 s, 72 °C—30 s	386
	Reverse	TCT CCA AGA CAC ATT ATC T		
*IL-1β* (16176)	Forward	GAC CTT GGA TGA GGA CA	95 °C—15 s; 60 °C—30 s, 72 °C—30 s	183
	Reverse	AGC TCA TAT GGG TCC GAC AG		
*Gapdh* (14433)	Forward	CCG CAG CGA GGA GTT TCT C	95 °C—15 s; 60 °C—30 s, 72 °C—30 s	530
	Reverse	GAG CTA AGC TCA GGC TGT TCC A		

ID = gene identification number, bp = base pairs.

## Data Availability

Data will be made available on request.

## Supplementary Materials

The following supporting information can be downloaded at: https://www.mdpi.com/article/10.3390/ani16060955/s1. Figure S1. Evaluation of T lymphocytes in the peritoneal lavage fluid of mice treated with (+) or without (−) cyclophosphamide (Cy) and crotoxin (CTX) and infected with (+) or without (−) *Encephalitozoon cuniculi*. (A) Percentage of CD4^+^ T lymphocytes. (B) Median fluorescence intensity (MFI) of IFN-γ in CD4^+^ T lymphocytes. (C) Percentage of CD8^+^ T lymphocytes. (D) MFI of IFN-γ in CD8^+^ T lymphocytes. Data were compared using one-way analysis, followed by Tukey’s post hoc test, *p* < 0.05 *, *p* < 0.01 **. Figure S2. Evaluation of splenic macrophages in mice treated with (+) or without (−) cyclophosphamide and crotoxin and infected with (+) or without (−) *Encephalitozoon cuniculi*. (A) Percentage (%) of Itgam^+^Adgre1^+^ macrophages. (B) Median fluorescence intensity (MFI) of CD80 and CD86 in Itgam^+^Adgre1^+^ macrophages. (C) MFI of IL-12a in Itgam^+^Adgre1^+^ macrophages. Data were compared using one-way analysis of variance, followed by Tukey’s post hoc test, *p* < 0.05 *.

## Abbreviations

The following abbreviations are used in this manuscript:

CTX	crotoxin
Cy	cyclophosphamide
