# Supplementary figures and images for: Crotoxin Upregulating NLRP-3 Inflammasome and IL-18 and Activating CD4+ and CD8+ Lymphocytes in Experimental Encephalitozoon cuniculi Infection
Source: Animals (Basel). 2026 Mar 18;16(6):955. doi: 10.3390/ani16060955 (PMC13023292; doi:10.3390/ani16060955)

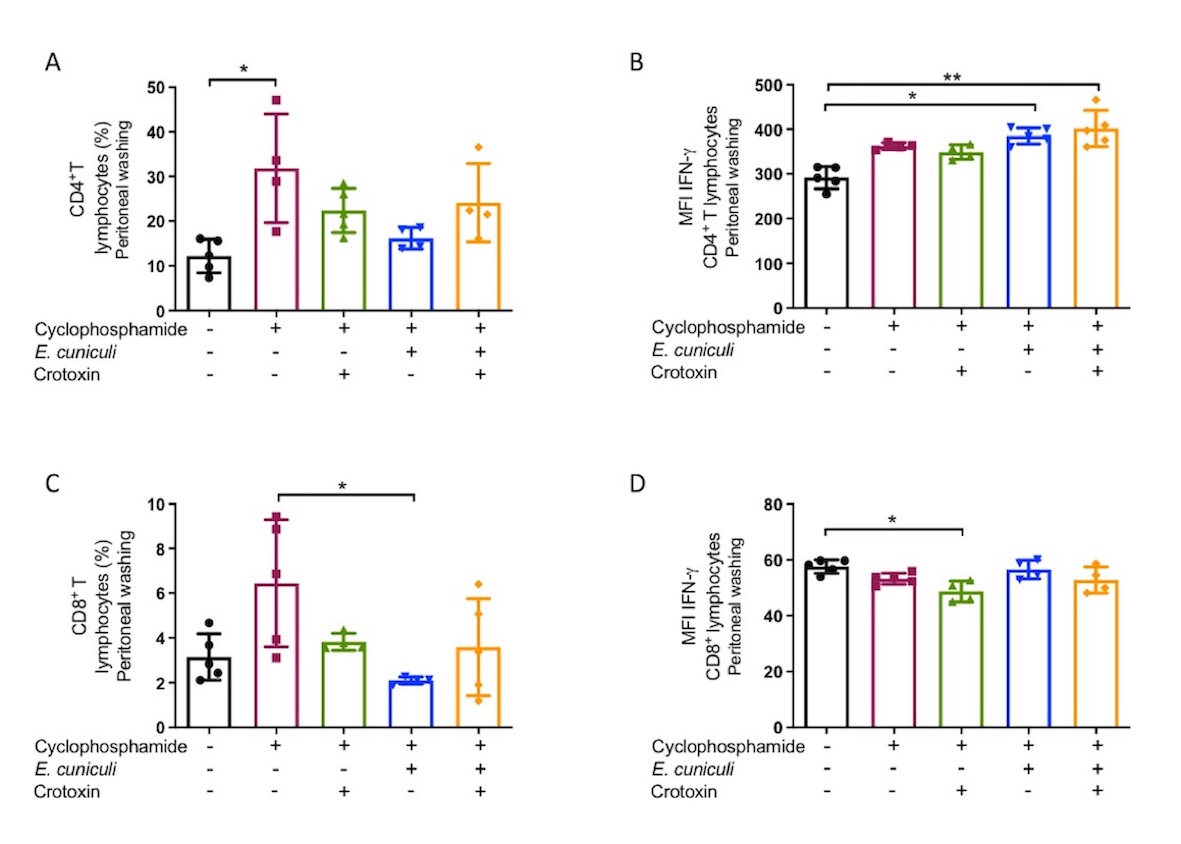

Supplement: Supplementary file 1 [file animals-16-00955-s001.zip › Figure S1.jpg]

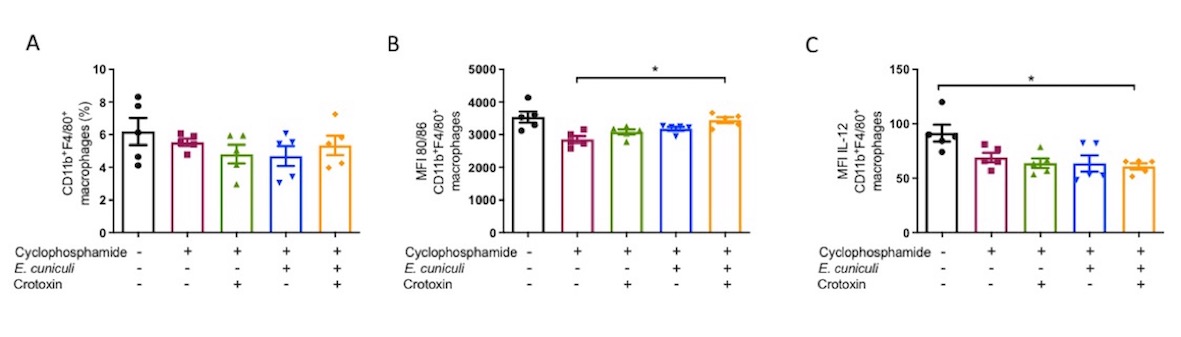

Supplement: Supplementary file 1 [file animals-16-00955-s001.zip › Figure S2.jpg]
